# 成人血液病患者新型冠状病毒疫苗接种中国专家共识（2023年版）

**DOI:** 10.3760/cma.j.issn.0253-2727.2023.01.004

**Published:** 2023-01

**Authors:** 中国康复医学会血液病康复专业委员会 中华医学会血液学分会

新型冠状病毒（以下简称“新冠病毒”）仍在全球范围内蔓延，据WHO统计，截至2022年12月19日，全球累计确诊COVID-19病例超过6.49亿，死亡人数逾664万，其中我国累计确诊病例1 007万，死亡3.13万（含港澳台地区，其中本土死亡5 235例）[1]。2022年初，中国康复医学会血液病康复专业委员会和中华医学会血液学分会共同组织专家撰写了《成人血液病患者接种新型冠状病毒疫苗中国专家共识（2022年版）》（以下简称“2022版共识”）[2]，为我国血液病患者进行新冠疫苗接种提供了重要指导。2022年以来，具有更强免疫逃逸和传播力的Omicron变异株已成为全球范围内的主要流行株，同时新冠病毒相关临床与基础研究取得了多方面的进展，新冠疫苗接种的重要性、安全性和有效性得到了进一步确认。根据疫情发展和Omicron变异株致病力下降的现状，国务院联防联控机制先后发布了优化疫情防控策略的“二十条”和“新十条”，表明我国疫情防控策略发生了巨大转变。为适应这一变化，使我国血液病患者更为合理有效地进行新冠疫苗接种及感染防控，现对2022版共识进行更新和修订，形成《成人血液病患者新型冠状病毒疫苗接种中国专家共识（2023年版）》，供同行参考。

建议一：血液病患者及其共同生活的家属和照护者需规范和严格执行戴口罩、勤洗手、勤通风、保持社交距离等非药物防护措施；血液病患者的家属和照护者如无禁忌，应接种新冠疫苗，并尽早完成加强免疫接种。

建议说明：

新冠病毒经过不断变异，受关注的各变异株（variant of concern，VOC）传播力和致病力也有所不同，Omicron变异株的免疫逃逸和传播力更强。现有证据表明，接种疫苗仍有重要的防重症和防死亡的保护作用，但防感染的效果不到20％。同时，随着疫苗接种完成后时间的推移，疫苗诱导的免疫保护效果不断下降，个体受感染的可能性逐渐增加[3]–[6]。研究表明，非药物性防护措施（戴口罩、勤洗手、勤通风、保持社交距离等）与接种新冠疫苗相结合，可有效减少新冠病毒的早期传播[7]。美国疾控中心对确诊病例的密接家人进行的调查显示，67.8％确诊病例家庭发生了聚集性感染，确诊病例家人的新冠确诊率达52.7％。其中，进行过加强接种、过去5个月内完成2剂基础疫苗接种以及未接种疫苗的家人，新冠确诊率分别为42.7％、43.6％和63.9％。确诊病例的家人如采取隔离措施，则新冠确诊率显著低于未采取隔离措施的家人（41.2％比67.5％）。在确诊患者的感染潜伏期，佩戴口罩的家人新冠确诊率显著低于从未佩戴口罩的家人（39.5％比68.9％）[8]。因此，建议血液病患者、家人及其照护者均需规范和严格执行非药物防护措施，血液病患者的家人及其照护者如无接种禁忌，均应完善新冠疫苗基础免疫，并尽早完成加强免疫接种。

建议二：建议符合疫苗接种条件的血液病患者均完善新冠疫苗接种。接种前应综合考虑疫情风险和患者的病情，充分评估接种疫苗的风险/获益，并遵循知情、自愿的原则。

建议说明：

一项基于上海Omicron变异株流行期间（2021年12月2日至2022年5月13日）61.2万余例3岁以上新冠病毒感染者真实世界数据的研究表明，我国本土广泛使用的灭活疫苗对重症/危重症的保护效果为88.6％，死亡保护效果为91.7％，接受了灭活疫苗同源加强者，重症保护效果可增加到92.7％，死亡保护效果增至95.9％，而腺病毒载体疫苗（单剂）对重症/危重症的保护效果为77.9％，提示接种2剂和3剂灭活疫苗对迄今为止的新冠病毒VOC所致的重症和死亡仍有较好的保护作用[9]。该研究与美国一项纳入1 060万居民的队列研究[10]和美国疾控中心公开数据[11]均说明，大规模接种新冠疫苗仍是目前新冠病毒感染所致重症和死亡防控的关键手段之一。

多数血液病患者是新冠病毒感染后重症及死亡的高风险人群。有研究表明，未接种疫苗的血液肿瘤患者感染Omicron新冠变异株后的死亡率与此前的VOC相似（约31％），但对已接种过新冠疫苗的血液肿瘤患者，Omicron变异株突破性感染的30 d死亡率降低到7.9％[2],[12]–[13]。因此，建议血液病患者在充分考虑自身情况及疫情风险后，符合接种条件者均应接种新冠疫苗。应当指出的是，血液病患者接种疫苗后，应加强不良反应的观察，同时注意对原发血液病的密切随访观察。对伴有血小板减少或有出血倾向的患者应注意疫苗注射部位的止血，有血栓形成倾向的患者应关注疫苗接种后血小板计数和出凝血指标的变化[2]。

建议三：血液病患者可优先选择灭活疫苗，亦可考虑使用重组亚单位疫苗。一般禁止使用减毒活病毒载体疫苗[2]。

建议说明：

截至2022年12月18日，我国累计接种新冠疫苗34.57亿剂次。据2022年7月23日国务院联防联控机制新闻发布会信息，在新冠疫苗接种不良反应报告中累计报告接种后不良事件238 215例，总体报告发生率为70.45/100万，其中一般反应发生率为57.27/100万（占不良反应的81.29％），严重异常反应发生率为0.64/100万（占不良反应的0.91％）。该不良反应报告率低于2020年我国接种其他常规疫苗报告的水平。WHO早在2020年5月制定的安全手册中就定义了新冠疫苗相关的特别关注不良事件（adverse event of special interest，AESI）。香港的一项研究显示，对于肿瘤患者，在肿瘤活动期接种灭活疫苗和未接种者的AESI发生率分别为0.13和0.88每10 000人天；在有既往肿瘤史的患者中，接种灭活疫苗和未接种者的AESI发生率分别为0.42和0.93每10 000人天。虽然这一研究可能存在一定的患者选择偏倚，即健康状况相对较好或预后较好的肿瘤患者更有可能接受疫苗接种，但仍然在一定程度上反映了新冠灭活疫苗在肿瘤人群中（包括血液肿瘤）的安全性[14]。重组亚单位疫苗的一项关键性研究显示，与安慰剂组相比，总不良事件和严重不良事件发生率相当，且98.5％为轻度不良反应；接种第2剂及第3剂疫苗（每剂间隔30 d）时，不良反应发生率未进一步增加；且≥60岁的接种者不良事件发生率低于18～59岁的接种者（28.8％比42.3％）[15]。

因此，基于既往同类型疫苗的安全性及目前新冠疫苗接种不良事件的报道[2]，建议血液病患者优先选择接种灭活疫苗，重组亚单位疫苗也可应用。如果后续国家有对新VOC针对性更强的疫苗可供时，建议参照使用。

建议四：满足疫苗接种条件的血液病患者，在完成疫苗基础免疫3个月后，可行1剂加强免疫接种，如果加强接种后已超过6个月，可考虑再行1剂加强接种。

建议说明：

现阶段国内外研究均指出，完成新冠疫苗基础免疫3个月后，疫苗的保护效力会随着时间的推移逐步降低，且对不断出现的病毒VOC保护效力下降，适时进行加强免疫接种可有效降低病毒VOC导致的症状性感染率和感染后的重症率及死亡率[9],[16]。不论是同源加强还是异源加强方案，都能在不同程度上提高免疫保护效力，而且不同的加强方案之间无显著差异[17]–[18]。研究表明在Omicron变异株流行时期，接种1剂加强疫苗的血液肿瘤患者发生突破性感染后，住院率及死亡风险仍是非血液肿瘤人群的5.6倍[19]。因此，血液肿瘤患者应适时再行加强疫苗接种。欧洲一项多中心研究显示，血液肿瘤患者接种第2剂加强免疫后，新冠重症/危重症率相比该系列研究既往报道数据显著降低[20]–[22]。以色列一项在Omicron变异株流行期间进行的全国性真实世界研究显示，在第1剂加强接种完成4个月后，接种第2剂加强可使60岁以上老年人近期（7～30 d）新冠感染风险降低55％，症状性感染风险降低45％，重症风险降低38％，死亡风险降低26％[23]。中国香港卫生署2021年12月31日至2022年10月12日期间（Omicron变异株流行时期）的数据显示，接种1、2、3、4剂灭活疫苗的人群总病死率分别为1.45％、0.35％、0.11％、0.04％，80岁以上老人的病死率分别为7.15％、3.75％、1.58％和0.71％，而该年龄组未接种疫苗的病死率高达14.76％。其中，4剂灭活疫苗对近期新冠相关死亡的保护效果与mRNA疫苗相同，均为96％，且加强接种并不增加疫苗相关不良事件的发生[24]。因此，在病毒变异不断演进的过程中，不仅1剂加强疫苗非常重要，尽早进行第2剂加强免疫也十分必要。

伴有免疫功能受损的血液病患者，接种新冠疫苗后体液及细胞免疫应答水平一般低于健康人群，加强免疫接种可提高免疫应答水平，大部分血液肿瘤患者接种1剂加强疫苗后可达到与健康人群接种2剂基础免疫相当的抗体应答水平[25]–[27]。随接种疫苗后时间的推移，血液病患者抗体滴度衰减的速率较健康人群更快，应适时再行加强免疫接种，以利于获得更高水平和更长时间的保护。因此，建议符合接种条件的血液病患者在完成基础免疫接种3个月后进行第1剂加强接种，完成加强接种6个月后，可行第2剂加强接种。基于推荐血液病患者接种的疫苗类型，结合国家卫健委2022年12月13日发布的《新冠病毒疫苗第二剂次加强免疫接种实施方案》的推荐，可采用灭活疫苗同源加强或灭活疫苗与重组亚单位疫苗的异源加强。

建议五：既往新冠感染康复的血液病患者，如符合接种条件，也应采取与无新冠感染史的血液病患者相同的疫苗接种策略。

建议说明：

巴西一项Omicron变异株流行前的回顾性研究发现，新冠感染康复者接种疫苗后，再次发生症状性感染的风险降低39.4％～64.8％，新冠相关死亡风险降低>80％[28]。瑞典一项对新冠感染康复者的全国性研究表明，与单纯自然感染获得的免疫保护效果相比较，接种1剂或2剂疫苗者在接种后2个月内发生再次感染的风险分别降低58％和66％，而接种2剂疫苗的保护效果能维持9个月以上；无论是接种1剂还是2剂疫苗，均可显著降低再次发生症状性感染患者的住院风险[29]，提示杂合免疫（自然感染+1或2剂疫苗）对新冠感染康复者具有长期保护效果。

既往非Omicron变异株自然感染所产生的免疫应答水平，将随感染者康复后时间的推移逐步降低，而Omicron变异株免疫逃逸能力更强，因此，单纯自然感染产生的免疫保护效力不足。一项病例对照研究表明，感染非Omicron变异株康复者再次发生Omicron BA.4或BA.5症状性感染的保护效果仅为35.5％，而感染Omicron株后产生的免疫应答对再次感染Omicron变异株的保护效果可达76.2％，但这一保护效果是否可以长期维持尚不清楚[30]–[32]。因此，对于血液病患者，即使既往有过新冠病毒感染史，仍建议在感染康复6个月后进行规范的疫苗接种，以降低再次感染的发生率。

建议六：接受造血干细胞移植（HSCT）或嵌合抗原受体T细胞（CAR-T细胞）治疗的患者，应根据疾病状态和造血及免疫功能恢复情况，在细胞回输3～6个月后接种新冠疫苗；治疗前接种过疫苗的患者，治疗后应重新进行全程疫苗接种；存在中、重度（Ⅱ～Ⅳ）或难治性急性移植物抗宿主病（GVHD）或广泛型慢性GVHD且尚在应用免疫抑制剂的患者，建议暂缓接种。

建议说明：

HSCT和CAR-T细胞治疗后造血和免疫功能恢复的程度与疫苗接种后的血清学反应密切相关。HSCT后至接种疫苗的间隔时间越长，患者的血清反应率越高，≤6个月、7～12个月以及>12个月的血清阳性率分别为38.2％，62.3％以及87.9％；而接受针对淋巴细胞或浆细胞抗原的CAR-T细胞治疗的患者，疫苗接种后的总体血清阳性率仅为35.9％。有报道免疫抑制剂的应用可降低疫苗接种后的血清学反应5.86倍，移植后患者淋巴细胞计数<1×10^9^/L者，疫苗接种的血清学反应可降低4.44倍[33]。虽然推迟治疗后接种疫苗的时间可能使患者获得更好的免疫应答，但其在细胞输注后免疫力低下，适逢高传播力的Omicron变异株流行，这一新冠感染高风险人群一旦感染，死亡风险可升高4.8倍[34]。有研究表明，淋巴细胞计数在HSCT后1～2个月内较低，3个月起逐渐恢复，开始重建部分免疫功能[35]，故最早可在HSCT或CAR-T细胞治疗3个月后进行新冠疫苗接种，此时机体可对疫苗产生一定程度的免疫应答。一项荷兰的前瞻性多中心研究显示，异基因HSCT后4个月接种新冠疫苗可产生有效的体液免疫应答，包括具有慢性GVHD的患者[36]。2022 NCCN指南也推荐HSCT和CAR-T受者最早可在细胞输注3个月后开始接种新冠疫苗[37]。综上，建议接受HSCT或针对淋巴细胞或浆细胞抗原的CAR-T细胞治疗的患者，应根据疾病状态和造血及免疫功能恢复情况，于细胞输注3～6个月后接种新冠疫苗。

在治疗之前接种过新冠疫苗的患者，因移植预处理及淋巴细胞清除等治疗措施使机体丧失既往疫苗诱导的免疫记忆细胞和特异性免疫防护力，故需在HSCT和CAR-T细胞治疗后重新进行新冠疫苗的全程接种。而存在中、重度（Ⅱ～Ⅳ）或难治性急性GVHD或广泛性慢性GVHD且尚在应用免疫抑制剂的患者，出于安全性和有效性考虑，建议暂缓接种新冠疫苗。

建议七：建议暂缓接种疫苗的其他情况：①ANC<1.0×10^9^/L、PLT<50×10^9^/L；②正在治疗中的免疫相关血细胞减少性疾病，如免疫性血小板减少症（ITP）、自身免疫性溶血性贫血、再生障碍性贫血（AA）、阵发性睡眠性血红蛋白尿症（PNH）等；③正在接受强化疗；④正在接受抗B淋巴细胞药物治疗。以下情况不建议接种疫苗：噬血细胞综合征；血栓性血小板减少性紫癜（TTP）；系统性肥大细胞增多症；血液病相关出凝血功能异常；存在国家卫生健康委员会“新冠病毒疫苗接种技术指南（第一版）”描述的疫苗接种禁忌证的患者[2]。

建议说明：

出于接种疫苗的安全性考虑，我们设定了血细胞初步恢复的参考指标（ANC≥1.0×10^9^/L、PLT≥50×10^9^/L）。此外，近2年陆续有疫苗接种后血液学相关不良反应的报道，使部分免疫相关血细胞减少性血液病患者能否接种疫苗及接种时机不易把握。英国药品与医疗保健产品监管机构收到16份来自mRNA新冠疫苗相关免疫性血小板减少症报告[38]；美国疫苗不良事件报告系统中有77例接种mRNA疫苗后1周左右出现的新发ITP，117例原发ITP患者接种第1剂或第2剂mRNA疫苗后，分别有19例和14例患者出现血小板显著下降，多见于脾切除或接受≥5线治疗的患者[39]；另有一项报告显示，32例有TTP既往史的患者，接种mRNA疫苗后有4例TTP复发[40]。因此，对于免疫相关血细胞减少性血液病患者，如正在进行原发病治疗且病情未获控制，可暂缓接种。对于疾病控制良好的患者，接种后需密切监测血常规等相关疾病指标。例如，ADAMTS13<20％的缓解期TTP患者，应在接种疫苗后2～4周内每周行血常规和ADAMTS13活性检测[41]。近期虽有研究显示重型AA、PNH患者接种mRNA疫苗可获得较好的免疫应答且安全性良好[42]–[43]，但鉴于目前仍缺乏大样本数据支持，尤其是病情未控制和正在进行免疫抑制相关治疗的此类患者，建议暂缓接种。此外，也有mRNA疫苗接种后出现AA[44]、获得性血友病[45]等病例报告，但无法明确这些罕见不良事件与疫苗接种的相关性。虽然这些疫苗相关的免疫性血细胞异常事件的报告多见于mRNA疫苗接种后，灭活疫苗等其他技术路径的疫苗也不能排除类似事件的发生，因此建议这类患者暂缓接种或不接种。噬血细胞综合征、系统性肥大细胞增多症等疾病因易于释放细胞因子或血管活性物质，在有新的证据前暂不建议接种，尤其是病情未获控制的患者。

建议八：建议暂缓接种或不接种新冠疫苗的患者以及接种后无法产生有效免疫应答的患者，如存在新冠病毒感染高危因素，可考虑应用长效中和抗体进行新冠病毒暴露前预防，但不可替代疫苗接种。

建议说明：

血液病的疾病种类、疾病阶段、治疗方案和接种时机均可影响疫苗接种后机体的免疫应答率和应答水平[2]。如接受自体HSCT的骨髓瘤患者，产生有效抗体应答的最短接种间期为移植后2个月；异基因HSCT的患者产生有效抗体应答的最短接种间期为移植后4～6个月[36]。因此，尽管通过改善疫苗接种策略可提高大部分血液病患者的疫苗保护效果，但对暂缓接种或不接种的患者以及接种后无法产生有效免疫应答的患者，当存在新冠病毒感染高危因素时，仍需要更多的暴露前预防措施以提供额外的保护，应用长效中和抗体进行被动免疫以提供即时保护，是重要途径之一。

替沙格韦单抗/西加韦单抗（AZD7442）是目前国际上唯一经Ⅲ期临床研究证实可用于新冠暴露前预防的中和抗体，使用后第8天和第29天的体内抗体水平分别是新冠病毒感染患者恢复期血浆样本的16倍和22倍，6个月期间可降低新冠病毒症状性感染风险82.8％，已被多个国家批准用于中重度免疫功能低下和对新冠疫苗接种免疫应答不佳的患者[46]，可降低Omicron变异株突破性感染风险49％, 降低住院/死亡风险92％[47]。目前，AZD7442正申请在我国上市的批文。2021年底我国已批准了一项国产新冠病毒中和抗体组合（罗米司韦单抗/安巴韦单抗）的临床应用，但其适应证为新冠感染后的治疗，对感染高风险患者是否有暴露前预防作用，目前尚无证据。

## 总结

新冠疫情已3年整，人们对该病毒特性及其致病性已有较为深入的认识，但由于其高突变率，至少已经有α、β、δ和Omicron等多个主要的VOC在不同国家、地区或全球流行，且已有新VOC流行的报道。虽然基于原始病毒株研制的疫苗对VOC感染的预防作用不佳，但数据显示至今仍有重要的防重症和防死亡作用。本版共识充分复习了现有的相关文献，引用了更多较大样本的研究结果，综合共识编撰专家的临床经验和意见，形成了对血液病患者疫苗接种和感染防控的主要建议，总结于图1，以供血液病工作者及相关专业的医务人员参考。鉴于当前国家新的防疫策略和疫情形势，血液病患者作为免疫功能特别脆弱的群体，感染风险较前大增，因此接种疫苗的建议较2022版共识更为积极。但由于血液病病种和病情的高度异质性，对于每一位患者，仍需结合具体情况，将接种、暂缓接种或不接种的所有建议综合考虑，充分评估接种疫苗的风险与获益，以获得最大可能的保护。今后如果国家有对流行VOC针对性更强的疫苗或抗体药物可供，建议参照使用。

**图1:**
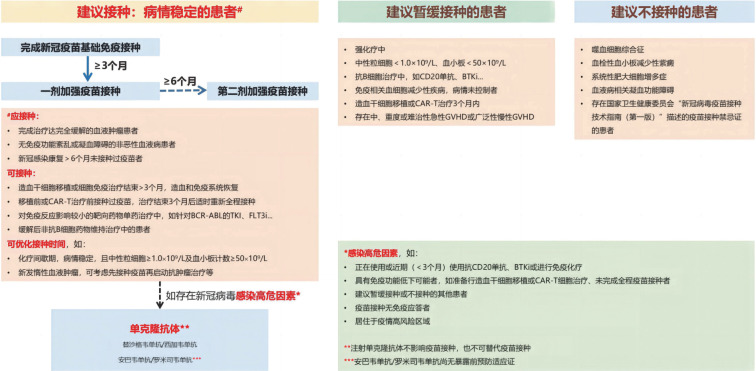
血液病患者COVID-19疫苗接种的主要建议
